# A Genomic Selection Index Applied to Simulated and Real Data

**DOI:** 10.1534/g3.115.019869

**Published:** 2015-08-18

**Authors:** J. Jesus Ceron-Rojas, José Crossa, Vivi N. Arief, Kaye Basford, Jessica Rutkoski, Diego Jarquín, Gregorio Alvarado, Yoseph Beyene, Kassa Semagn, Ian DeLacy

**Affiliations:** * Biometrics and Statistics Unit, International Maize and Wheat Improvement Center (CIMMYT), 06600, México Distrito Federal, México; † The University of Queensland, School of Agriculture and Food Sciences, St Lucia, QLD 4072, Brisbane, Australia; ‡ International Programs of the College of Agriculture and Life Sciences, Cornell University, Ithaca, New York 14853; § Department of Agronomy and Horticulture, University of Nebraska, Lincoln, Nebraska 68583; ** Global Maize Program, CIMMYT, Village Market 00621, Nairobi, Kenya

**Keywords:** genomic estimated breeding value, net genetic merit, selection index, selection response, genomic selection, GenPred, shared data resource

## Abstract

A genomic selection index (GSI) is a linear combination of genomic estimated breeding values that uses genomic markers to predict the net genetic merit and select parents from a nonphenotyped testing population. Some authors have proposed a GSI; however, they have not used simulated or real data to validate the GSI theory and have not explained how to estimate the GSI selection response and the GSI expected genetic gain per selection cycle for the unobserved traits after the first selection cycle to obtain information about the genetic gains in each subsequent selection cycle. In this paper, we develop the theory of a GSI and apply it to two simulated and four real data sets with four traits. Also, we numerically compare its efficiency with that of the phenotypic selection index (PSI) by using the ratio of the GSI response over the PSI response, and the PSI and GSI expected genetic gain per selection cycle for observed and unobserved traits, respectively. In addition, we used the Technow inequality to compare GSI *vs.* PSI efficiency. Results from the simulated data were confirmed by the real data, indicating that GSI was more efficient than PSI per unit of time.

In genomic selection (GS), phenotypic and marker data from the training population are fitted in a statistical model to estimate all available marker effects. These estimates can then be used in subsequent selection cycles to obtain genomic estimated breeding values (GEBVs) that are predictors of the breeding values in the testing population (candidates for selection) for which there is only marker information (Meuwissen *et al.* 2001; Heffner *et al.* 2009; Lorenz *et al.* 2011; Nakaya and Isobe 2012). In GS, GEBVs are tools for ranking and selecting candidates for selection. Bernardo and Yu (2007) and Heffner *et al.* (2011) have shown that selection based on genomic predictions can lead to greater genetic gains per unit of time for complex traits. Technow *et al.* (2013) derived an inequality that depends on GS accuracy and the square root of the heritability of the unobserved trait, which is useful to compare the genomic selection efficiency with the phenotypic efficiency in terms of time.

The standard method for predicting marker effects and breeding values is the ridge-regression best linear unbiased predictor, or its equivalent, the genomic best linear unbiased predictor, which assumes that the effects of all markers have a multivariate normal distribution with mean zero and constant variance (Meuwissen *et al.* 2001; VanRaden 2008). The difference among the various Bayesian regression methods lies in how they specify the prior distribution of the parameters of interest (de los Campos *et al.* 2013; Gianola 2013). Methods such as Bayes A and Bayes B assume that the variance of marker effects has an *a priori* inverse χ^2^ distribution (Meuwissen *et al.* 2001) that produces shrinkage as well as variable selection. Nevertheless, when the true marker effects have a multivariate normal distribution and the size of the training population and the number of markers is large, all methods produce GEBVs that are highly correlated with the true breeding values of the candidates for selection (Hayes *et al.* 2009; Verbyla *et al.* 2010).

In the context of molecular marker-assisted selection, Lande and Thompson (1990) proposed a selection index that combines marker information with phenotypic information, whereas Dekkers (2007) proposed a selection index that combines GEBVs with phenotypic information. Both selection indices were evaluated using simulated data and in both studies the authors found that the estimated selection response was greater than when only phenotypic information was used to estimate it. In the context of GS, Togashi *et al.* (2011) proposed four selection indices similar to Dekkers’ index based on the best linear unbiased predictor theory; however, their results are hypothetical because the authors did not use any data (either simulated or real) to validate these indices. The indices of Togashi are a direct application of the phenotypic selection index (PSI) (Smith 1936), but they do not explain how to estimate the GS response and the genomic selection index (GSI) expected genetic gain per selection cycle for unobserved traits after the first selection cycle, which is important, because they give information on the genetic gains in the next selection cycle and are the base criteria to compare the efficiency of two or more linear selection indices (Bulmer 1980; Moreau *et al.* 1998).

This study had three main objectives: (1) to apply the GSI to two simulated and four real data sets that only use GEBVs for selecting nonphenotyped candidates for selection; (2) to propose a method to estimate the GSI selection response and the GSI expected genetic gain per selection cycle for unobserved traits after the first selection cycle; and (3) to compare GSI efficiency *vs.* PSI efficiency using simulated and real data.

## Materials and Methods

### PSIs and GSIs

The objective of any linear selection index, whether phenotypic or genomic, is to predict the net genetic merit $\text{H}=w^{\prime }a$, where $a^{\prime }=[\begin{array}{cccc} a_{1} & a_{2} & ... & a_{t} \end{array}]$ (*t* = number of traits) is a vector of true breeding values for an individual and $w^{\prime }=[\begin{array}{cccc} w_{1} & w_{2} & ... & w_{t} \end{array}]$ is a vector of economic weights. According to Kempthorne and Nordskog (1959), the selection response of any linear selection index can be written as

$$R=\frac{k}{L}\frac{\sigma_{H,I}}{\sigma_{I}^{2}}=\frac{k}{L}\sigma_{H}\rho_{H,I}$$ (1)

where *k* is the standardized selection differential (or selection intensity), $\sigma_{H,I}$ is the covariance between H and any linear index *I*, $\sigma_{I}^{2}$ is the variance of *I*, $\sigma_{H}$ is the standard deviation of H, $\rho_{H,I}$ is the correlation between H and any linear index *I*, and *L* denotes the time required to collect information to evaluate *I* and complete one selection cycle. The second part of Equation (1) ($\frac{k}{L}\sigma_{H}\rho_{H,I}$) indicates that the genetic change due to selection is proportional to $\rho_{H,I}$ and to *k*, which is the selection differential in the index in standard deviation units (Kempthorne and Nordskog 1959). If *k*, $\sigma_{H}$, and *L* are fixed, *R* will be maximized when $\rho_{H,I}$ is maximized and the final form of Equation (1) will depend on the particular linear selection index used to select individuals, *e.g.*, PSI or GSI.

#### PSI and its selection response:

Let $p^{\prime }=[\begin{array}{cccc} p_{1} & p_{2} & \ldots & p_{t} \end{array}]$ be a vector of phenotypic trait values; the PSI (Smith 1936) can be written as $\text{PSI}=b^{\prime }p$ and its maximized selection response is

$$R_{PSI}=\frac{k}{L_{PSI}}\sqrt{b^{\prime }Pb}$$ (2)

where $L_{PSI}$ denotes the time required for PSI to complete one selection cycle, $b=P^{-1}Cw$, $P^{-1}$ is the inverse of the phenotypic covariance matrix (**P**), and **C** is the covariance matrix of true breeding values **a**; *k* and **w** were defined previously.

#### GSI and its selection response:

The GSI can be written as

$$\text{GSI}=w^{\prime }\gamma$$ (3)

where $\gamma^{\prime }=[\begin{array}{cccc} {\text{γ}}_{1} & {\text{γ}}_{2} & ... & {\text{γ}}_{t} \end{array}]$ is a 1×t vector of genomic breeding values for one individual; it can be shown that the maximized GSI selection response is

$$R_{GSI}=\frac{k}{L_{GSI}}\sqrt{w^{\prime }\Gamma w}$$ (4)

where $L_{GSI}$ denotes the time required for GSI to complete one selection cycle; *k* and **w** were defined previously; $\Gamma =\{\sigma_{\gamma qq^{\prime }}\}$ ($q,q^{\prime }=\text{1,2,}...\text{,t}$) is a covariance matrix of additive genomic breeding values ***γ***.

Note that in each selection cycle, matrices **P**, **C**, and ***Γ*** change their values as a result of many individuals being eliminated by the selection process.

#### Estimating the parameters of the PSI:

In each selection cycle, we used the restricted maximum likelihood method (Patterson and Thompson 1971) to estimate the covariance matrix of true breeding values (**C**) and of the residuals (**R**), which were denoted as $\hat{C}$ and $\hat{R}$, respectively, from where matrix $\hat{P}=\hat{C}+\hat{R}$ was an estimator of the phenotypic variance-covariance matrix (**P**). We estimated $b=P^{-1}Cw$ and $R_{PSI}=\frac{k}{L_{PSI}}\sqrt{b^{\prime }Pb}$ as $\hat{b}={\hat{P}}^{-1}\hat{C}w$ and ${\hat{R}}_{PSI}=\frac{k}{L_{PSI}}\sqrt{\hat{b^{\prime }}\hat{P}\hat{b}}$, respectively.

#### Estimating the GEBV and the GSI in the l^th^ selection cycle:

Let $\hat{u}$ be the estimator of the vector of marker effects $u^{\prime }=[\begin{array}{cccc} {u^{\prime }}_{1} & {u^{\prime }}_{2} & \ldots & {u^{\prime }}_{t} \end{array}]$ for *t* traits (Appendix). We obtained the $q^{th}$ GEBVs (q=1,2,…,t) in the $l^{th}$ selection cycle (l=1,2,...,number of cycles) as

$${\hat{\text{γ}}}_{ql}=X_{l}{\hat{u}}_{q}$$ (5)

where ${\hat{u}}_{q}$ is the vector of size m×1 of the marker effects of the $q^{th}$ trait in the base population and $X_{l}$ is a matrix of size g×m of the coded values of marker genotypes in the $l^{th}$ selection cycle (Goddard 2009). The estimated GSI (**GSI**_E_) values in this cycle were

$$GSI_{\text{E}}=\sum_{q=\text{1}}^{\text{t}}w_{q}{\hat{\gamma }}_{q}{}_{l}$$ (6)

where $w_{q}$ is the $q^{th}$ economic weight and ${\hat{\text{γ}}}_{ql}$ was defined in Equation (5). Note that Equation (6) is a vector of size g×1 (*g* = number of genotypes). In practice, **GSI**_E_ values are ranked to select individual genotypes with optimum GEBV values.

#### Estimating the ***Γ*** matrix:

Suppose that $\gamma_{q}$ and $\gamma_{q^{\prime }}$ have multivariate normal distribution jointly, with mean $1\mu_{\gamma_{q}}$ and $1\mu_{\gamma_{q^{\prime }}}$, respectively, and covariance matrix $G\sigma_{\gamma_{qq^{\prime }}}$, where **1** is a g×1 vector of 1s and $G=XX^{\prime }/c$ is the additive genomic relationship matrix (Appendix). Then $\Gamma =\{\sigma_{\gamma_{qq^{\prime }}}\}$ can be estimated as

$${\hat{\Gamma }}_{l}=\{{\hat{\sigma }}_{\gamma_{qq^{\prime }}}\}$$ (7)

where ${\hat{\sigma }}_{\gamma_{qq^{\prime }}}=\frac{1}{g}({\hat{\text{γ}}}_{ql}-1{\hat{\mu }}_{\gamma_{ql}}{)}^{\prime }G_{l}^{-1}({\hat{\text{γ}}}_{q^{\prime }l}-1{\hat{\mu }}_{\gamma_{q^{\prime }l}})$ is the estimated covariance between $\gamma_{q}$ and $\gamma_{q^{\prime }}$ in the $l^{th}$ selection cycle; *g* is the number of genotypes; ${\hat{\text{γ}}}_{ql}$ was defined in Equation (5); ${\hat{\mu }}_{\gamma_{ql}}$ and ${\hat{\mu }}_{\gamma_{q^{\prime }l}}$ are the estimated arithmetic means of the values of ${\hat{\text{γ}}}_{ql}$ and ${\hat{\text{γ}}}_{q^{\prime }l}$; **1** is a g×1 vector of 1s and $G_{l}=c^{-1}X_{l}{X^{\prime }}_{l}$ is the additive genomic relationship matrix in the $l^{th}$ selection cycle (l=1,2,..., number of cycles). From Equations (4) and (7), the estimated GSI response is ${\hat{R}}_{GSI}=\frac{k}{L_{GSI}}\sqrt{w^{\prime }{\hat{\Gamma }}_{l}w}.$

### Criteria for comparing GSI efficiency *vs.* PSI efficiency

Assuming that *k* is the same in both indices, to compare GSI efficiency *vs.* PSI efficiency in the $l^{th}$ selection cycle, we used the ratio

$$\lambda =\frac{{\hat{R}}_{GSI}}{{\hat{R}}_{PSI}}=\frac{L_{PSI}}{L_{GSI}}\sqrt{\frac{w^{\prime }{\hat{\Gamma }}_{l}w}{\hat{b}‘{\hat{P}}_{l}\hat{b}}}=\frac{L_{PSI}}{L_{GSI}}\frac{{\hat{\rho }}_{H,GSI}}{{\hat{\rho }}_{H,PSI}}$$ (8)

which was proposed by Bulmer (1980) and Moreau *et al.* (1998) as a criterion for comparing the efficiency of linear selection indices. In Equation (8), ${\hat{R}}_{PSI}$ and ${\hat{R}}_{GSI}$ are estimators of Equations (2) and (4), respectively, and ${\hat{\rho }}_{H,GSI}$ and ${\hat{\rho }}_{H,PSI}$ are the maximized estimated correlation (or accuracy) between H and GSI, and between H and PSI, respectively. Using this criterion, if λ>1, GSI efficiency will be greater than PSI efficiency, if λ=1, the efficiency of both selection indices will be equal, and if λ<1, PSI will be more efficient than GSI.

### PSI and GSI expected genetic gain per selection cycle

Besides Equation (8) for comparing the efficiency of PSI *vs.* GSI, we used the estimated values of the following two equations:

$$E_{PSI}=\frac{k}{L_{PSI}}\frac{Cb}{\sqrt{b^{\prime }Pb}}$$ (9)

$$E_{GSI}=\frac{k}{L_{GSI}}\frac{\Gamma w}{\sqrt{w^{\prime }\Gamma w}}$$ (10)

where **E***_PSI_* and **E***_GSI_* are the expected genetic gain per selection cycle for each trait in the PSI (Lin 1978) and in the GSI (Togashi *et al.* 2011), respectively. All the terms in Equations (9) and (10) were defined and estimated according to Equations (2) and (4), respectively.

### Simulated and real data sets

#### Simulated data sets (data sets 1 and 2):

**Figure 1:**
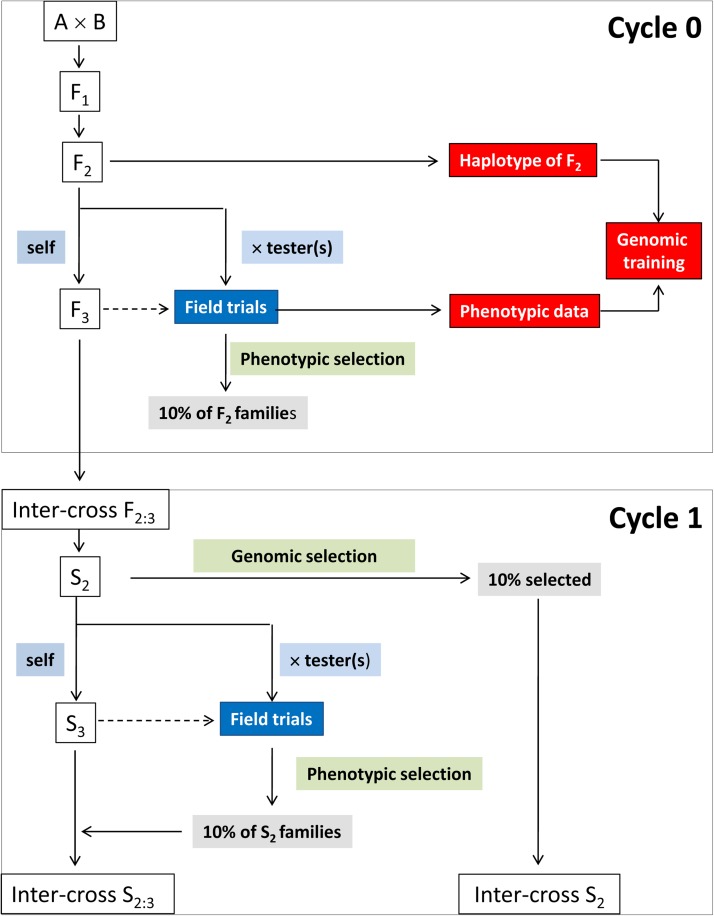
Schematic illustration of the steps followed to generate data sets 1 and 2 for the selection process using the phenotypic selection index and the genomic selection index. Dotted lines indicate the process used to simulate the phenotypic data.

Figure 1 presents a schematic illustration of the steps followed to generate the simulated data sets. For the simulation, the performance of the *F*_2_ or *S_n_* families was evaluated using the selfing generation (*F*_3_ or *S_n_*_+1_) of the *F*_2_ or *S_n_* families, whereas in practice, the *F*_2_ or *S_n_* families would be evaluated by crossing them to a tester (or testers).

We simulated eight phenotypic selection cycles [cycle 0 (C0)−cycle 7 (C7)] for PSI (data set 1), and seven GS cycles (C1−C7) for GSI (data set 2), each with four traits (T1, T2, T3, and T4), 500 genotypes and four replicates for each genotype under one possible scenario: 5% of quantitative trait loci (QTL) were in linkage equilibrium.

C0 was the GSI training population, which contained phenotypic and genotypic data; it is the population where we estimated the molecular marker effects (Appendix). In all selection cycles, we selected and intermated the top 10% of individuals (k=1.75). The economic weights used in PSI and GSI for T1, T2, T3, and T4 were 1, −1, 1, and 1, respectively. Selections were based on PSI and GSI values that incorporated all four trait (T1, T2, T3, and T4) means in each selection cycle to predict and select the net genetic merit ($\text{H}=w^{\prime }a$) of each individual.

Simulated data were generated using QU-GENE software (Podlich and Cooper 1998; Wang *et al.* 2003). Three hundred fifteen QTL and 2500 molecular markers were distributed uniformly across 10 chromosomes to simulate two maize (*Zea mays* L.) populations. Each QTL and molecular marker was biallelic and the QTL additive values ranged from 0 to 0.5. The 315 QTL were randomly allocated over the 10 chromosomes. Because QU-GENE uses recombination fraction rather than map distance to calculate the probability of crossover events, recombination between adjacent pairs of markers was set at 0.0906, those between a QTL and its flanking markers set at 0.0 and 0.0906, and that between two adjacent QTL set at 0.0. The recombination fraction between 15 random QTL and their flanking markers was set at 0.5, *i.e.*, complete independence (Haldane 1919), to simulate linkage equilibrium between 5% of the QTL and their flanking markers.

Each of the four traits (T1, T2, T3, and T4) was affected by a different number of QTL: 300, 100, 60, and 40, respectively. The common QTL affecting the traits generated genotypic correlations of −0.5, 0.4, 0.3, −0.3, −0.2, and 0.1 between T1 and T2, T1 and T3, T1 and T4, T2 and T3, T2 and T4, and T3 and T4, respectively.

The genotypic value of each plant was generated based on its haplotypes and the QTL effects for each trait. For each trait, the phenotypic value for each of four replications of each plant was obtained from QU-GENE software by setting the per-plot heritability of T1, T2, T3, and T4 at 0.4, 0.6, 0.6, and 0.8, respectively.

In cycle C0 (the training population), 500 *F*_2_ plants were generated from a cross of two inbred parents. The haplotypes of these parents were randomly generated, but the two parents shared no common alleles. In subsequent cycles (*i.e.*, C1−C7), 500 plants were generated from a random intercross of the selected 10% of lines from the previous cycle using the PSI and GSI methods. In C0, only PSI was applied. In C1, two selection methods were applied: PSI (data set 1) and GSI (data set 2); the 10% of individuals selected with each method were advanced to the next selection cycle.

#### Real data sets 3, 4, 5, and 6:

These data sets (data sets 3, 4, 5, and 6) correspond to four maize (*Zea mays* L.) *F*_2_ populations. They were used by Beyene *et al.* (2015) and were denoted as JMpop1 DTMA Mexico optimum environment, JMpop1 DTMA Zimbabwe optimum environment, JMpop3 DTMA Mexico optimum environment, and 6x1020 WEMA Africa optimum environment, respectively. These data were used to perform GS in eight biparental populations; field evaluation of a training population (C0), cycle 1 (C1), and cycle 2 (C2) from GS was reported by Beyene *et al.* (2015).

For each data set, C0 contained genotypic data and four phenotypic traits: grain yield (GY, t/ha), plant height (PHT, cm), ear height (EHT, cm), and anthesis days (AD, d), as well as three sets of markers corresponding to C0 (training population), C1, and C2. The numbers of individuals and molecular markers in each population are shown in Table 1. Assuming that the breeding objective was to increase GY while decreasing PHT, EHT, and AD, the vectors of economic weights in C0, C1, and C2 for GY, PHT, EHT, and AD, were $w^{\prime }=[\begin{array}{cccc} 5 & -0.3 & -0.3 & -1 \end{array}]$ for both indices and the four data sets.

**Table 1:** Four real maize (*Zea mays* L.) ${\text{F}}_{\text{2}}$ populations and the number of individuals (*i*) and molecular markers (*m*) used in one PSI selection cycle (cycle 0) and in three GSI selection cycles (cycles 0, 1, and 2)

Cycle	Real Data Sets							
	3		4		5		6	
	*i*	*m*	*i*	*M*	*i*	*M*	*i*	*m*
0	247	195	247	195	234	190	181	205
1	320	195	320	195	396	190	274	205
2	303	195	303	195	269	190	274	205

PSI, phenotypic selection index.

In our study, the PSI was applied only in C0 because there were no phenotypic data after that cycle, whereas GSI was applied in C0, C1, and C2. Note that GSI was used in C0 only with the purpose of comparing GSI efficiency *vs.* PSI efficiency. The top 10% (*k* = 1.75) was selected in all cycles of the four data sets.

We analyzed the simulated and real data results for all traits in each selection cycle, by using three different criteria: the estimated GSI and PSI selection responses, the estimated expected genetic gain per selection cycle for each trait in the PSI and in the GSI, and the estimated Technow *et al.* (2013) inequality (see Supporting Information, File S1 for the last criteria).

#### Data repository:

The simulated phenotypic selection cycles (C0−C7) for PSI (data set 1), and GS cycles (C1−C7) for GSI (data set 2), as well as the real data sets (data sets 3−6) including the phenotype and haplotype data are deposited at http://hdl.handle.net/11529/10199. This repository also has the File S1 cited several times in the text of the paper.

### Data availability

The data repository has the following data: *Real_Data_Sets_GSI, Simulated_Data_GSI,* and a manuscript*: Supplementary Material-2.doc* that are described below

File *Real_Data_Sets_GSI* contains four file data sets: DATA_SET-3, 4, 5 and 6. In addition, each four file data sets contains four excel data. For example, the four excel data for file DATA_SET-3 are: DATA_SET-3_Markers_Cycle-0, 1, 2, and DATA_SET-3_Phenotypic_Cycle-0. The first three excel data contains the marker coded values for cycles 0, 1 and 2, while the excel data DATA_SET-3_Phenotypic_Cycle-0 contains the phenotypic information of cycle 0 (Training population). These four data sets were used to make selection, to estimate the selection response and the genetic expected gains; the results were presented in Table 4.

The other three file data sets: DATA_SET-4, 5 and 6 contains similar information that file DATA_SET-3, but this information correspond to data set 4, 5 and 6 used to make selection, to estimate the selection response and the genetic expected gains; the results were presented in Table 4.

File *Simulated_Data_GSI* contains two files: *Data_Phenotypes_April-26-15* and *Haplotypes_GSI_April-26-15*. File *Data_Phenotypes_April-26-15* contains two files: GSI_Phenotypes-05 and PSI_Phenotypes-05. File GSI_Phenotypes-05 contains six excel data sets denoted as C2_GSI_05_Pheno, C3_GSI_05_Pheno, C4_GSI_05_Pheno, C5_GSI_05_Pheno and C6_GSI_05_Pheno, corresponding to the phenotypic simulated information for genomic selection index for cycle 2-7, meanwhile GSI_Phenotypes-05 contains eight excel data sets denoted as *C0_Pheno_05, C1_PSI_05_Pheno, C2_PSI_05_Pheno, C3_PSI_05_Pheno, C4_PSI_05_Pheno, C5_PSI_05_Pheno, C6_PSI_05_Pheno, C7_PSI_05_Pheno* corresponding to the phenotypic simulated information for phenotypic selection index for cycle 0-7. File Haplotypes_GSI_April-26-15 contains the haplotypes of the markers for cycles 0-7 of GSI.

Finally, the manuscript *Supplementary Material-2.doc* contain a complete description related with the form we adapted the Technow inequality to the genomic and phenotypic selection index to comparer its efficiency in terms of time. In addition, this manuscript contains Table S1 and Table S2; the first one contains the results of the simulated data and the second one the results of real data.

## Results

### Simulated data

#### Correlations between GEBV and the trait true breeding values:

**Figure 2:**
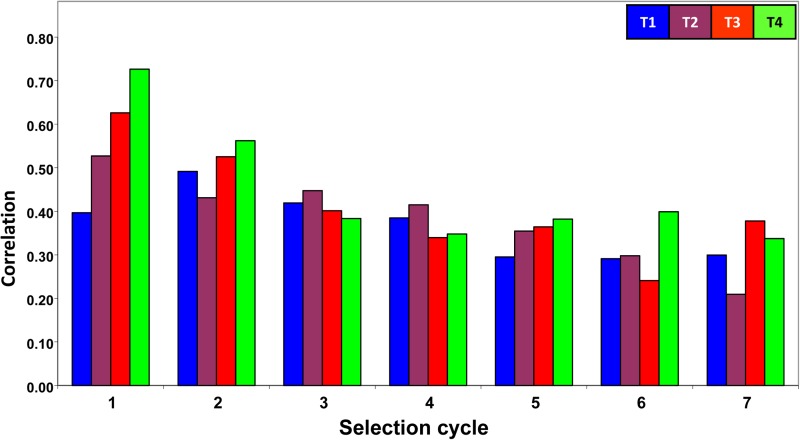
Correlation between the genomic estimated breeding values (GEBVs) and the true breeding values for four traits in seven selection cycles. For each cycle of selection, the four columns correspond to the correlation between the GEBV and the true breeding values for traits T1, T2, T3, and T4, respectively.

Figure 2 shows the correlation between the GEBV and the individual trait true breeding values obtained by the Pearson correlation coefficient. The genomic relationship (**G**) is not incorporated in the correlations. In Figure 2, each selection cycle contains four columns: the first column (from left to the right) corresponds to the correlation between the GEBV and the T1 true breeding values; the second column corresponds to the correlation between the GEBV and the T2 true breeding values; etc. In this figure, all correlation values tend to decrease after the first selection cycle. In C7, the correlation values between the GEBV and the trait true breeding values were 0.30, 0.21, 0.38, and 0.34, for each of the four traits, respectively, whereas in cycle one (C1) these correlations were 0.40, 0.53, 0.63, and 0.73, for each of the four traits, respectively. In terms of proportions, the correlation values of C7 were only 76%, 40%, 60%, and 46% of the correlation values of C1. That is, the correlation between the GEBV and the trait true breeding values decreased more for traits 2 and 4 than for traits 1 and 3. This can be explained by the number of QTL that affected each trait and the size of the QTL effects on the traits in each selection cycle.

#### Correlations between the GSI and the true H values:

**Figure 3:**
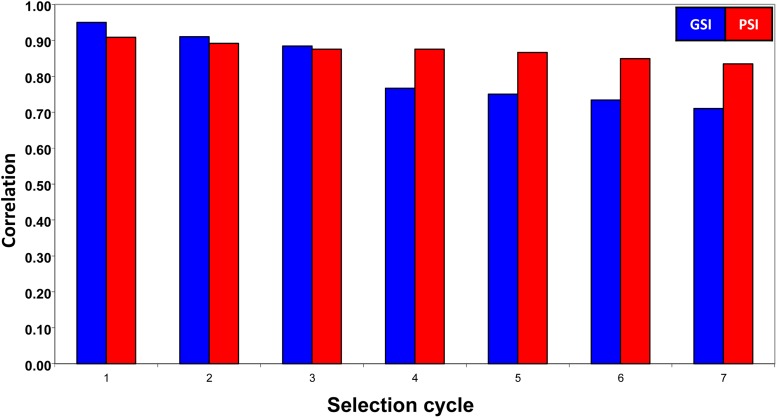
Correlation between the genomic selection index (GSI), the phenotypic selection index (PSI), and the true net genetic merit (H) values in seven selection cycles. For each cycle of selection, the first column corresponds to the correlation between the GSI estimated values and the H true values (blue), whereas the second column corresponds to the correlation between the PSI estimated values and the H true values (red).

Figure 3 shows the correlation between the GSI and the true net genetic merit values ($\text{H}=w^{\prime }a$), for the four simulated traits in seven selection cycles. This correlation is computed as $\rho_{GSI,H}=\frac{\sqrt{w^{\prime }\hat{\Gamma }w}}{\sqrt{w^{\prime }Cw}}$, where **C** is the covariance matrix of true breeding values (Equation (1)) and $\hat{\Gamma }$ was obtained according to Equation (7). In this case, $\rho_{GSI,H}$ incorporated the genomic relationship (**G**) information.

Figure 3 contains only two columns for each selection cycle: the first column (blue) corresponds to the correlation between the GSI and the true values of H, whereas the second column (red) denotes the correlation between PSI estimated values and H. As expected, the correlation between GSI and H tended to decrease more than the correlation between PSI and H after the third selection cycle. The reason was that the PSI estimated values in each selection cycle were obtained using all phenotypic information of the newly generated population, whereas the GSI estimated values in each selection cycle incorporated only the marker information of the newly generated population. The correlation between GSI and H was 0.71 in C7 and 0.95 in C1, whereas the correlation between PSI and H was 0.83 in C7 and 0.91 in C1.

#### Estimated and true selection response of PSI and GSI when their generation interval is ignored:

The first part of Table 2 shows the GSI estimated (${\hat{R}}_{GSI}$) and true ($R_{GSI}$), and the PSI estimated (${\hat{R}}_{PSI}$) and true ($R_{PSI}$) selection responses and their ratios: ${\hat{R}}_{GSI}$/$R_{GSI}$and ${\hat{R}}_{PSI}$/$R_{PSI}$, when their generation interval was ignored, for simulated data sets 1 and 2, respectively, for four traits (T1, T2, T3, and T4) and seven GSI and PSI selection cycles. In all selection cycles, ${\hat{R}}_{GSI}<{\hat{R}}_{PSI}$ and $R_{GSI}<R_{PSI}$. In addition, results indicated that, in general, ${\hat{R}}_{GSI}<R_{GSI}$ and ${\hat{R}}_{PSI}<R_{PSI}$, *i.e.*, ${\hat{R}}_{GSI}$ and ${\hat{R}}_{PSI}$, underestimated the $R_{GSI}$ and $R_{PSI}$ values in all selection cycles.

**Table 2:** Estimated (${\hat{R}}_{GSI}$) and true ($R_{GSI}$) GSI responses; estimated (${\hat{R}}_{PSI}$) and true ($R_{PSI}$) PSI responses, and the ratios: ${\hat{R}}_{GSI}$/$R_{GSI}$ and ${\hat{R}}_{PSI}$/$R_{PSI}$, when their generation intervals were ignored

Cycles	${\hat{R}}_{GSI}$	$R_{GSI}$	${\hat{R}}_{PSI}$	$R_{PSI}$	${\hat{R}}_{GSI}$/$R_{GSI}$	${\hat{R}}_{PSI}$/$R_{PSI}$
Generation intervals ignored						
1	14.40	13.26	17.80	19.63	1.09	0.91
2	13.91	15.28	15.72	17.56	0.91	0.90
3	13.61	15.37	14.20	16.49	0.89	0.86
4	12.30	16.05	14.32	16.32	0.77	0.88
5	11.40	15.17	13.60	15.99	0.75	0.85
6	10.61	14.49	12.00	14.69	0.73	0.82
7	11.21	15.82	11.60	14.90	0.71	0.78
Average	12.49	15.06	14.18	16.51	0.83	0.86

Cycles	${\hat{R}}_{GSI}$	$R_{GSI}$	${\hat{R}}_{PSI}$	$R_{PSI}$	${\hat{R}}_{GSI}$/${\hat{R}}_{PSI}$	$R_{GSI}$/$R_{PSI}$
Generation intervals included						
1	9.60	8.84	4.45	4.91	2.16	1.80
2	9.27	10.19	3.93	4.39	2.36	2.32
3	9.07	10.25	3.55	4.12	2.55	2.49
4	8.20	10.70	3.58	4.08	2.29	2.62
5	7.60	10.11	3.40	4.00	2.24	2.53
6	7.07	9.66	3.00	3.67	2.36	2.63
7	7.47	10.55	2.90	3.73	2.57	2.83
Average	8.32	10.04	3.54	4.13	2.36	2.46

Estimated (${\hat{R}}_{GSI}$) and true ($R_{GSI}$) GSI responses; estimated (${\hat{R}}_{PSI}$) and true ($R_{PSI}$) PSI responses, and the ratios: ${\hat{R}}_{GSI}$/${\hat{R}}_{PSI}$ and $R_{GSI}$/$R_{PSI}$, when their generation intervals were included in simulated data sets 1 and 2, respectively, for four traits (T1, T2, T3, and T4). We conducted eight selection cycles (including cycle 0) with PSI and seven (from cycle 1−7) with GSI. The average responses and ratio values from cycle 1 to 7 are shown in the last line of each sub-table. GSI, genomic selection index; PSI, phenotypic selection index

The average values for all selection cycles of ratios ${\hat{R}}_{GSI}$/$R_{GSI}$ and ${\hat{R}}_{PSI}$/$R_{PSI}$ were equal to 0.83 and 0.86, respectively, which indicated that ${\hat{R}}_{GSI}$ explained 83% of $R_{GSI}$ performance, whereas ${\hat{R}}_{PSI}$ explained 86% of $R_{PSI}$ performance. Then, in terms of mean values, the results indicated that ${\hat{R}}_{GSI}$ and ${\hat{R}}_{PSI}$ were good estimators of $R_{GSI}$ and $R_{PSI}$ performance, respectively. The main results here show that for each selection cycle, the estimated PSI response to selection was always higher than the true and estimated GSI response when the generation interval was not incorporated in the estimated selection response.

#### Estimated and true selection response of PSI and GSI when their generation interval is included:

The second part of Table 2 shows the GSI estimated (${\hat{R}}_{GSI}$) and true ($R_{GSI}$), and the PSI estimated (${\hat{R}}_{PSI}$) and true ($R_{PSI}$) selection response values and their ratios: ${\hat{R}}_{GSI}$/${\hat{R}}_{PSI}$ and $R_{GSI}$/$R_{PSI}$, when their generation interval was included, for simulated data sets 1 and 2 for four traits (T1, T2, T3, and T4) for seven GSI and PSI selection cycles. In this case, the time required to complete one GSI selection cycle was $L_{GSI}=1.5$ years, whereas for one PSI selection cycle it was $L_{PSI}=4$ years. According to the ratio values ${\hat{R}}_{GSI}$/${\hat{R}}_{PSI}$ and $R_{GSI}$/$R_{PSI}$, in all selection cycles GSI was more than twice as efficient as PSI. Then, when the generation interval of the estimated GSI and PSI selection response was included in the estimate response to selection, GSI was more efficient than the PSI in all selection cycles for the estimated and true selection responses.

#### Expected genetic gains for PSI and GSI in simulated data with and without generation interval:

Columns 2−9 (from left to right) in Table 3 show the PSI estimated expected genetic gains for each trait per selection cycle for the observed traits (Equation 9) and columns 10−17 show the estimated GSI expected genetic gain for each trait per selection cycle for the unobserved traits (Equation 10). Note that the PSI estimated expected genetic gains of columns 2−5 were not divided by 4 (the time required to collect information to evaluate PSI and complete one selection cycle). Similarly, the GSI estimated expected genetic gains of columns 9−13 were not divided by 1.5 (the time required to collect information to evaluate GSI and complete one selection cycle). When the generation interval was not considered, the expected value of PSI for traits in each cycle was always higher than the expected values of the GSI for those traits. However, per unit of time, the expected genetic gains of GSI (columns 14–17 of Table 3) for each cycle and for each trait were always higher than the expected genetic gains of PSI (columns 6−9 of Table 3).

**Table 3:** Estimated expected genetic gains obtained using the PSI and the GSI for simulated data sets 1 and 2, respectively, for four traits (T1, T2, T3, and T4), when the generation interval is ignored and when it is included*^a^*

Cycles	PSI Estimated Expected Genetic Gains								GSI Estimated Expected Genetic Gains							
	Generation Interval Ignored				Generation Interval Included				Generation Interval Ignored				Generation Interval Included			
	T1	T2	T3	T4	T1	T2	T3	T4	T1	T2	T3	T4	T1	T2	T3	T4
1	7.9	−4.7	3.3	1.9	2.0	−1.2	0.8	0.5	6.6	−3.5	2.7	1.6	4.4	−2.3	1.8	1.1
2	7.1	−3.6	3.2	1.9	1.8	−0.9	0.8	0.5	6.3	−3.4	2.6	1.5	4.2	−2.3	1.7	1.0
3	6.7	−3.2	2.8	1.5	1.7	−0.8	0.7	0.4	6.1	−3.3	2.7	1.5	4.1	−2.2	1.8	1.0
4	7.5	−3.5	2.1	1.3	1.9	−0.9	0.5	0.3	5.6	−3.1	2.3	1.3	3.7	−2.1	1.5	0.9
5	7.1	−2.7	2.5	1.3	1.8	−0.7	0.6	0.3	5.2	−2.8	2.1	1.3	3.5	−1.9	1.4	0.9
6	6.2	−2.6	2.0	1.2	1.6	−0.7	0.5	0.3	4.9	−2.6	1.9	1.3	3.3	−1.7	1.3	0.9
7	5.4	−2.5	2.5	1.2	1.4	−0.6	0.6	0.3	5.2	−2.7	2.1	1.2	3.5	−1.8	1.4	0.8
Average	6.8	−3.3	2.6	1.5	1.7	−0.8	0.7	0.4	5.7	−3.1	2.3	1.4	3.8	−2.0	1.6	0.9

We conducted eight selection cycles (including cycle 0) with PSI and seven (from cycle 1 to 7) with GSI. The average responses and genetic gains from cycle 1 to 7 are shown in the last line of the table. PSI, phenotypic selection index; GSI, genomic selection index.

a For PSI, the time required to complete one selection cycle is 4 years; for GSI, the time required to complete one selection cycle is 1.5 years.

### Real data (F2 maize populations)

#### Estimated expected genetic gains and selection responses for PSI and GSI with generation interval included:

Table 4 shows the estimated PSI and GSI expected genetic gains, and the estimated PSI and GSI selection responses (Equations 2 and 4, respectively) for one PSI selection cycle (C0) and three GSI cycles (C0, C1, and C2) of four maize (*Zea mays* L.) $F_{\text{2}}$ populations and four traits (GY, PHT, EHT, and AD), when their generation interval was included. We aimed to increase GY while decreasing PHT, EHT, and AD by using three sets of markers (Table 1). As for the simulated data, for the PSI and GSI response, the time required to complete one selection cycle was $L_{GSI}=1.5$ and $L_{PSI}=4$ years for GSI and PSI, respectively.

**Table 4:** Expected genetic gains per selection cycle for the PSI and GSI for cycle 0 and cycles 0, 1, and 2, respectively, for four traits (GY, EHT, PHT, and AD) in four maize (*Zea mays*) ${\text{F}}_{\text{2}}$ populations when the generation interval was included

Traits	Data Set 3				Data Set 4			
	PSI Cycle	GSI Cycles			PSI Cycle	GSI Cycles		
	0	0	1	2	0	0	1	2
GY, kg/ha	102.5	153.8	137.8	120.4	195.0	550.7	471.4	461.4
EHT, cm	−1.73	−4.03	−3.43	−3.30	−1.16	−3.10	−2.63	−2.57
PHT, cm	−0.70	−4.30	−3.65	−3.73	−0.46	−1.18	−1.02	−0.99
AD, d	−0.04	−0.10	−0.04	−0.10	1.50	4.10	3.50	3.41
PSI or GSI responses	1.57	3.37	2.85	2.80	1.33	4.09	3.49	3.41

	Data Set 5				Data Set 6			
GY, kg/ha	320.5	401.5	433.9	285.6	93.1	222.4	230.1	194.7
EHT, cm	−2.09	−7.71	−0.75	−4.69	−0.57	−1.77	−1.15	−1.21
PHT, cm	−1.01	−0.34	−2.54	−0.78	−0.90	−1.65	−0.96	−1.07
AD, d	2.43	3.72	3.34	2.67	0.90	2.38	1.90	1.78
PSI or GSI responses	2.04	8.62	3.33	2.47	1.88	6.92	1.90	1.78

The last line of each subtable shows the estimated PSI (cycle 0) selection response, and the estimated GSI (cycles 0, 1, and 2) selection responses. PSI, phenotypic selection index; GSI, genomic selection index; GY, grain yield; EHT, ear height; PHT, plant height, AD, anthesis days.

#### Estimated expected genetic gains for PSI and GSI:

In this case, the GSI estimated expected genetic gain for each trait per selection cycle for the unobserved traits in C0 (or training population) were greater than the PSI estimated expected genetic gains for each trait per selection cycle for the observed traits. These results showed a similar tendency to the simulated results when the generation interval was included. That is, in C0, the estimated GSI expected genetic gains were greater than the estimated PSI expected genetic gains. In C1 and C2, it was not possible to compare GSI *vs.* PSI because there were no phenotypic data in those cycles.

#### Estimated PSI and GSI selection response:

The numbers of individuals and markers used in the four real data sets were lower (Table 1) than those used in the simulated data; for this reason, the estimated selection values observed in the real data sets (Table 4) were lower than those in the simulated results shown in Table 4. However, in general, the decrease in estimated GSI responses after C0 was similar to the decrease in estimated GSI selection responses after C1 in the simulated data (Table 2). For the real data sets, in C0, the estimated GSI selection response was higher than the estimated PSI selection response, whereas in C1 and C2, it was not possible to compare GSI *vs.* PSI because there were no phenotypic data in those cycles.

### Additional criteria for comparing PSI *vs.* GSI

Besides Equations (8), (9), and (10), we used the Technow *et al.* (2013) inequality adapted to the context of PSI and GSI (Supplemental Materials, Equation (S1)) as additional criteria to compare the efficiency of GSI *vs.* PSI in terms of time. This last criterion corroborated the results obtained with Equations (8), (9), and (10). Results of the last criterion are given in Table S1 and Table S2 for simulated and real data, respectively.

## Discussion

### Simulated data

Our results showed that GSI is more efficient than PSI per unit of time but not in terms of cycle. The average of the PSI and GSI selection responses values for all cycles, and the average of the PSI and GSI expected genetic gains per selection cycle for all cycles for observed and unobserved traits, respectively, were very similar when their generation interval was ignored because in the simulation process 95% of the QTL were in linkage disequilibrium with markers. After C3, the correlation between true and estimated PSI and GSI values was greater for PSI than for GSI. In our simulation, if instead of using 95% of the QTL in linkage disequilibrium, we had used 100% of the QTL in linkage disequilibrium with markers, we would expect the PSI and GSI results to be practically equal under the assumption of a very large number of markers. The importance of this result is that when the generation interval was ignored, PSI efficiency > GSI efficiency, but on average across all cycles, they were similar. When the interval length was used in the PSI and GSI selection responses and in the PSI and GSI expected genetic gain per selection cycle, GSI was always more efficient than PSI in maize population selection for relatively dense molecular markers in an *F*_2_ population.

We compared the PSI response with the GSI response considering the time (years) needed for each method to complete a selection cycle assuming that selection intensity is the same in both selection indices. Then, the ratio of the GSI selection response over the PSI selection response (Equation 8) was a good criterion for comparing PSI efficiency *vs.* GSI efficiency because each selection response included all the information on the genetic gains for each selection index in each selection cycle. In the case of the maize populations, GSI led to greater rates of genetic gain/year than PSI because PSI requires about 4 years to complete each selection cycle, whereas GSI requires about 1.5 years (Beyene *et al.* 2015). Thus GSI efficiency was greater than PSI efficiency because the interval of time between selection cycles in GSI is shorter than in PSI. If this factor is not taken into account, the average PSI response for the simulated data were 14% greater than the average GSI response.

### Real data

In the real data sets, the trend of GSI responses was very similar to those observed in the simulated data when their generation interval was not ignored. That is, GSI responses were higher than PSI responses in C0 for all four data sets (Table 4). One reason for these results may be that markers were in linkage disequilibrium with many QTL of the trait. In that case, GSI was very effective. As shown by Beyene *et al.* (2015), in eight biparental populations, a good genetic gain is expected from rapid cycling of GS in an *F*_2_ population with maximum linkage disequilibrium. Note that the estimated selection response for GSI decreased in a manner similar to that of the simulated data after cycle 0. This is because in the real data sets, the estimated selection response depends on the additive genomic variance-covariance matrix (***Γ***), whose covariance components decreased in each selection cycle.

### The importance of the estimation of matrix ***Γ*** in simulated and real data and its effect on GSI correlations, GSI response, and GSI expected genetic gains

We proposed one way of estimating matrix $\Gamma =\{\sigma_{\gamma_{qq^{\prime }}}\}$ (Equation 7). This method significantly affected (1) the correlation between GSI and the net genetic merit ($\text{H}=w^{\prime }a$), (2) the estimated GSI response, and (3) the estimated GSI expected genetic gains. The elements of ***Γ*** were estimated as ${\hat{\sigma }}_{\gamma_{qq^{\prime }}}=\frac{1}{g}({\hat{\gamma }}_{ql}-1{\hat{\mu }}_{\gamma_{ql}}{)}^{\prime }G_{l}^{-1}({\hat{\gamma }}_{q^{\prime }l}-1{\hat{\mu }}_{\gamma_{q^{\prime }l}})$. Another form of $\sigma_{\gamma_{qq^{\prime }}}$ estimate is ${\hat{\sigma }}_{\gamma_{qq^{\prime }}}=\frac{1}{g}({\hat{\gamma }}_{ql}-1{\hat{\mu }}_{\gamma_{ql}}{)}^{\prime }({\hat{\gamma }}_{q^{\prime }l}-1{\hat{\mu }}_{\gamma_{q^{\prime }l}})$, where matrix $G_{l}^{-1}$ is omitted. In that case, the correlation between the GSI and H would tend to be smaller (data not shown) than that shown in Figure 3. In addition, we could also expect that the estimated GSI selection responses and the estimated GSI expected genetic gains per selection cycle would be smaller than those shown in Table 2, Table 3, and Table 4.

These results indicate the importance of matrix $G_{l}^{-1}$ in the estimation of the GSI response, the GSI expected genetic gains per selection cycle, and in the correlation between GSI and H because the use of the genomic relationship matrix increases the accuracy of parameter estimation.

### PSI *vs.* GSI

PSI and GSI are predictors of H and both have optimal statistical properties. However, while PSI is a phenotypic predictor of H, GSI is a genomic predictor of H. Based on trait hereditability and genetic architecture, PSI is expected to be more accurate and have a greater selection response per selection cycle than GSI. However, in terms of genetic gain per unit of time, GSI needs one-third of the time required by PSI or less (Lorenz *et al.* 2011). Thus, GSI will be more efficient than PSI in GS programs. We have shown (in File S1) that the Technow inequality corroborated this last argument.

In simulation and empirical studies, GEBVs based solely on individual genotypes have been remarkably accurate. These accuracies depend on the characteristics of the population under selection (Lorenz *et al.* 2011). According to Equations (3) and (6), GSI is a linear combination of indices because GEBVs are indices *per se* (Robinson 1991; Togashi *et al.* 2011) and its main function is to predict the net genetic merit ($\text{H}=w^{\prime }a$) of the candidate for selection. According to classical best linear unbiased predictor theory (McLean *et al.* 1991; Robinson 1991): (a) GSI is the best linear predictor of H; (b) the correlation between GSI and H is maximum; (c) the GEBVs are unique; and (d) $E(\text{H}/{\text{GSI}}_{\text{E}})={\text{GSI}}_{\text{E}}$, *i.e.*, the expectation of H given GSI_E_ is GSI_E_. PSI was constructed with trait phenotypic means to predict and select H; however, Henderson (1963) showed that all four points are also true for PSI when matrices **P** and **C** are known.

For the selection objective, GSI requires only the genomic best linear unbiased predictor obtained in the training population (in this case, C0) and the population markers of each selection cycle that are used to obtain the GEBV in each selection cycle. Then, for selection proposes, we only need to construct the estimated GSI as $GSI_{\text{E}}=\sum_{q=\text{1}}^{\text{t}}w_{q}{\hat{\gamma }}_{q}{}_{l}$ (Equation 6) and the $GSI_{\text{E}}$ values are then ranked to select individual genotypes with optimum GEBV values. However, in the present paper, we used the PSI theory originally developed by Smith (1936) to obtain the GSI selection response and the GSI expected genetic gains per selection cycle. Selection response and expected genetic gains give information on the genetic gains in the next selection cycle and are the base criteria for comparing the efficiency of two or more selection indices (Bulmer 1980; Moreau *et al.* 1998).

### PSI *vs.* GSI when the generation interval is equal in both indices

Some of the results shown in Tables 2 and Table 3 and Table S1 and Table S2 occurred when the PSI generation interval (*L_PSI_*) was greater than the GSI generation interval (*L_PSI_*). What would happen if *L_PSI_* = *L_GSI_*? In this case, if the number of markers is very small, then Equation (4) will give lower values than Equation (2) and PSI efficiency will be greater than GSI efficiency. However, if the number of markers is very large, the PSI and GSI responses will be very similar.

This argument also holds true for the Technow *et al.* (2013) inequality and the PSI and GSI expected genetic gain per selection cycle for observed and unobserved traits. For example, note that in Table S1, we have assumed that $L_{GSI}=1.5$ and $L_{PSI}=4.0$. Suppose now that $L_{PSI}=L_{GSI}=4.0$. In this case, the Technow *et al.* (2013) inequality will not hold true because in all selection cycles $L_{GSI}>\frac{\rho_{H,GSI}}{h_{PSI}}L_{PSI}$. That is, the Technow *et al.* (2013) inequality will change its direction. Finally, it is evident that if $L_{PSI}<L_{GSI}$, PSI will be more efficient than GSI even in the hypothetical case when the number of molecular marker is infinite. In conclusion, GSI will be more efficient than PSI in terms of unit of time only if $L_{PSI}>L_{GSI}$; in this case, the Technow *et al.* (2013) inequality is true. In all other cases, PSI will be more efficient than GSI.

In this study, we applied the theory of GSI to simulated and real data and compared its efficiency with PSI efficiency by using three different criteria: the ratio of the GSI response over the PSI response, the PSI and GSI expected genetic gain per selection cycle for observed and unobserved traits, respectively, and the Technow inequality. In all three cases, for simulated and real data, GSI efficiency was higher than PSI efficiency per unit of time in all selection cycles. We thus concluded that GSI is an efficient choice when the purpose of a breeding program is to select individuals using GS.

## Appendix

### Multivariate prediction of molecular marker effects

In the univariate context, VanRaden (2008) showed that marker effects in the training population could be estimated as

$${\hat{u}}_{q}=c^{-1}X^{\prime }{\left[G+\lambda I_{g}\right]}^{-1}y_{q}$$ (A1)

where $G=c^{-1}XX^{\prime }$ is the additive genomic relationship matrix, **X** is a matrix with coded marker values on the base population (*e.g.*, 1, 0, and −1 for genotypes *AA*, *Aa*, and *aa*, respectively); *p_j_* is the allelic frequency of *a*; $c=\sum_{j=1}^{N}2p_{j}(1-p_{j})$ is a proportional constant (Habier *et al.* 2007); $\lambda =\frac{\sigma_{eq}^{2}}{\sigma_{aq}^{2}}$; $\sigma_{aq}^{2}$ and $\sigma_{eq}^{2}$ are the additive and residual variances, respectively, associated with the *q*^th^ trait; $y_{q}$ ∼ NMV(**0**, $V_{q}$) is a vector of observations, where NMV stands for the multivariate normal distribution, $V_{q}=G\sigma_{\text{a}q}^{2}+I_{g}\sigma_{e_{q}}^{2}$; and $I_{g}$ is an identity matrix of order g×g.

In the multivariate context, to estimate the vector $u^{\prime }=[\begin{array}{cccc} {u^{\prime }}_{1} & {u^{\prime }}_{2} & \ldots & {u^{\prime }}_{t} \end{array}]$, Equation (A1) can be written as

$$\hat{u}=c^{-1}{Z^{\prime }}_{t}{\left[\left(I_{t}⊗G)+(L⊗I_{g}\right)\right]}^{-1}y$$ (A2)

where $Z_{t}=I_{t}⊗X$, “⊗” denotes the direct product, **X** was defined in Equation (A1); $L=RC^{-1}$, **R** is the residual covariance matrix, and **C** was defined in the text (Equation 1); $y^{\prime }=[\begin{array}{cccc} {y^{\prime }}_{1} & {y^{\prime }}_{2} & \ldots & {y^{\prime }}_{t} \end{array}]$ ∼ NMV(**0**, ***V***) is a vector of size 1×tg, and $V=C⊗G+R⊗I_{g}$; $I_{t}$ is an identity matrix of size t×t and $I_{g}$ was defined in Equation (A1). In this case, the estimator of the vector $\gamma^{\prime }=[\begin{array}{cccc} \gamma_{1} & \gamma_{2} & ... & \gamma_{t} \end{array}]$ is $\hat{\gamma }=Z_{t}\hat{u}$.
